# A shot in the foot: Could chemical control of malaria vectors threaten food security?

**DOI:** 10.5281/zenodo.13969756

**Published:** 2024-10-22

**Authors:** Bart G.J. Knols

**Affiliations:** 1 Editor, MalariaWorld Journal

## Abstract

Overwhelmingly, contemporary malaria vector control equals the use of chemical pesticides (through insecticide-treated bednets or indoor residual spraying). Gradually, but surely, we have become enslaved to thinking that controlling malaria mosquitoes equals the use of chemical insecticides, and much of the vector control field today is dominated by scientists, lobbyists, chemical companies, funding agencies and (global) institutions that endlessly repeat this dogmatic belief. Although chemical control has undoubtedly saved millions of lives, which, morally speaking would immediately justify its continued use, it has many sides that may ultimately cost more lives than it saves. Not only the cyclical problems with insecticide resistance, but also our increased understanding of the human and environmental health impacts of these chemicals, continue to raise red flags. Furthermore, the millions of kilogrammes of annual bednet waste (polyethylene, polypropylene) and bednet packaging material cannot be ignored. In recent years, an abundance of evidence that the use of chemical pesticides is a prime cause for the global decline in insect biodiversity and abundance has surfaced. The rate at which this decline is happening is frightening and may sooner rather than later threaten food production on a global scale. Should we opt for saving lives in the short term by using chemicals and face devastating and irrevocable long-term consequences or become wise(r) in the way we control malaria mosquitoes?

In 2022, Dr Pedro Alonso, the former Director of the WHO Global Malaria Programme, commented:

*“Taken together, we are facing a malaria crisis. We need to do things differently. As Einstein famously said, doing the same thing over and over again and expecting a different result is insanity”* [1].

His message followed the disturbing reality that the global reduction in both malaria cases and deaths, which had been so encouraging between 2000 and 2015, had been plateauing for the past seven years. Since then, nothing much has changed.

The crisis that Alonso referred to knows many causes. Read any opening paragraph of a malaria article these days and it will list all the problems nicely for you. Resistance to drugs and insecticides, not enough financial resources, failing diagnostics, etc. It is more the second part of Alonso’s statement that intrigues me: That if you continue to do what you did, you will end up with what you got (which is also one of Einstein’s famous quotes).

Recently, I posted a comment on LinkedIn that read as follows:

*“To anyone who works on insecticides, be it for indoor residual spraying, be it for bednet impregnation, please read this book by Oliver Milman. Insect biodiversity is undergoing a massive decline around the world, yet this is largely ignored. We have perfect biological alternatives to control mosquitoes that transmit diseases, yet these are not given a chance by large chemical companies (Bayer, Syngenta, BASF, etc.) or the World Health Organization, IVCC (Innovative Vector Control Consortium) etc. It is no longer a choice if we should change the way we do things in vector-borne disease control, it is a must. #malaria #pestcontrol #biological #biodiversity”*

**Figure:**
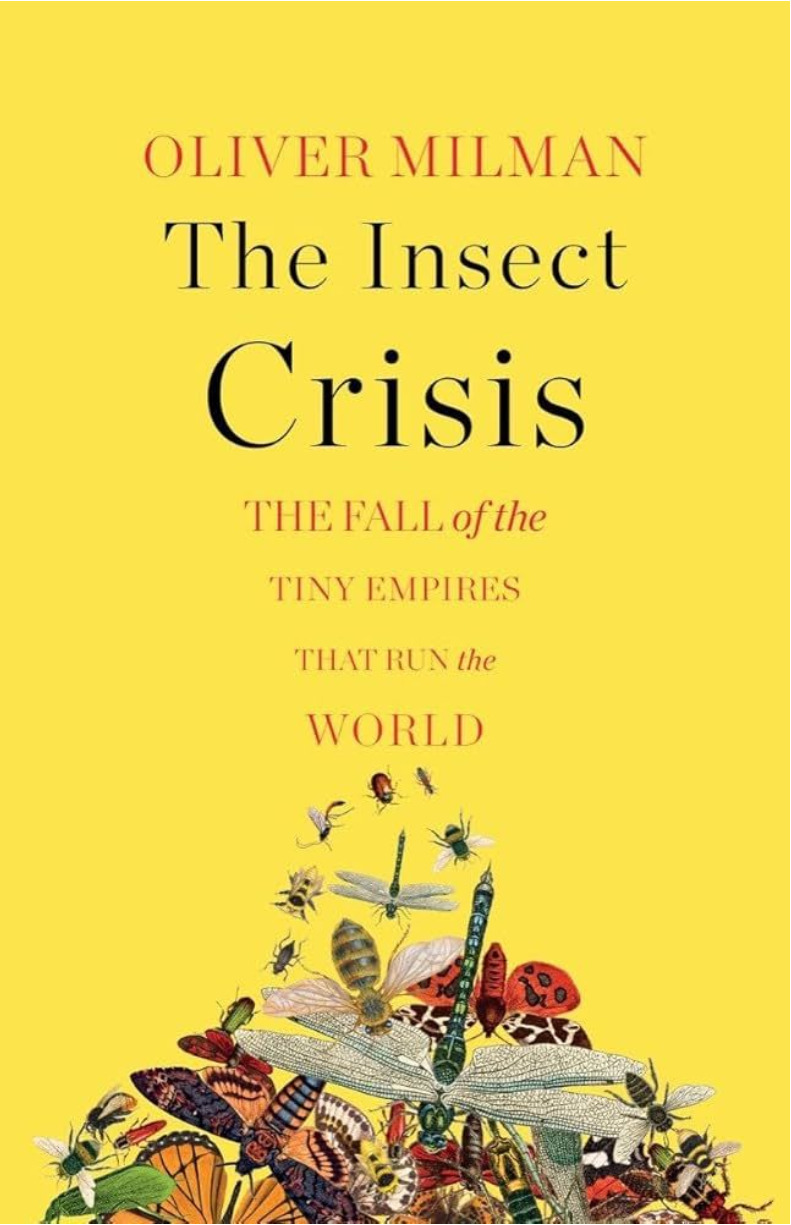


Like many of us, I post things on LinkedIn from time to time, but this time the response was unexpectedly high. Clearly, this message struck readers. And although I have been known to argue against chemical pesticides for years (due to my upbringing at Wageningen University in the Netherlands, which has a strong focus on biological control of insect pests), this time the response was far more remarkable.

Little did I know that two days prior to my post, an article came online, led by scientists from my former University, that rang alarm bells on the likely impact of broad-scale neonicotinoid use in agriculture on bird populations around the world [2]. Not just because these chemicals kill sources of food for birds (insects), but also because of increasing evidence that these chemicals directly impact bird health, behaviour, reproduction and survival. Although the authors mention the difficulty of discerning direct, indirect and delayed effects, they call for caution in using existing and new neonicotinoids. This was the next article in a long list of papers that focus on the negative human, animal and environmental health impacts of this class of chemicals; the mostly broadly used in the world today.

It is of course ‘easy’ to blame agriculture for this. The use of chemical pesticides for vector control indeed dwarfs in comparison to their use in agriculture. Besides this, it is often argued that we ‘stay away’ from non-target insects because in malaria control we use chemical pesticides indoors, either through indoor residual spraying (IRS) or long-lasting insecticide treated bednets (LLINs). This was one of the reasons through which DDT ‘survived’ a global ban being part of the POPs treaty [3].

Yet, although nets are (mostly) used indoors, we seem to forget (or ignore) what happens to them when no longer used. Although nets have been developed to last for three years, the average lifespan of a net is far below that, studies have shown [4]. A recent UNITAID report calculated that bednet waste will reach the level of 57,5 million kilograms annually by 2030 [5]. That’s 57,5 million kilograms of plastic (polyethylene or polypropylene), much of the yarn still holding actives (pyrethroids, chlorfenapyr, piryproxyfen, PBO or combinations thereof) that end up outdoors and can leak chemicals into the environment. Of the more than 3 billion nets that have been distributed in sub-Saharan Africa, the vast majority came in plastic bags, resulting in direct waste. In 2014, WHO published guidelines for the proper disposal of old nets but did not progress beyond recommending incineration in high-temperature furnaces (largely absent in most areas where nets are used), or burial (in non-permeable soil) [6]. It is more likely that old nets simply end up in the environment or are burned in open fires resulting in the release of highly toxic fumes (such as dioxines). As for IRS, it has been shown that indoor sprayed chemicals do not remain in the domestic environment but rather exit sprayed houses in the form of house-dust and may be persistent in the surrounding environment for a long time afterwards [7,8]. Clearly, the arguments used in the past no longer hold: Intensification of chemical malaria vector control results in millions of kilograms of plastic, laden with insecticide, in the environment and indoor spraying clearly leads to these chemicals ending up outdoors. So we can’t just put the blame on agriculture. Chemical-based malaria vector control nowadays occurs at such large scale that introspection on our side is called for and necessary.

But search ‘environmental impact, indoor residual spraying, malaria’ in PubMed, and there is nothing much to find on how IRS is having an impact on insects other than its target (the mosquito). If anything, we know that pyrethroid-treated nets have resulted in highly resistant strains of bedbugs having great impact on people’s comfort at night [9]. But you’ll find nothing on how insect biodiversity compares between villages sprayed with IRS chemicals and those not being sprayed. We pretty much measure the impact of our actions on malaria vectors and parameters such as malaria incidence, and that’s where it stops. We’re in the game of controlling, and even better, eliminating malaria and we’re not interested (or even competent) in insect taxonomy and the conservation of some butterfly, bumble bee or dragonfly in the surroundings of houses where children die of malaria. The question is: should we?

Being Dutch, this is easy for me to say of course. In my country (The Netherlands) nobody suffers any longer from malaria, let alone die from it (Holland was declared malaria-free in 1970). And it is of course up to every endemic country to make its own risk/benefit analyses of using chemicals. However, and this is the big however, my country (as is the rest of Europe) is currently experiencing what has been termed the ‘insectageddon’ (derived from the word armageddon). The loss in insect diversity is staggering according to recent studies and have a cascading effect in our ecosystems with insectivorous predators (e.g., swallows and bats) also declining at frightening speeds. Some call this the forebode of the total collapse of global biodiversity. Anyone who reads Oliver Milman’s book ‘The Insect Crisis’ [10] will be shocked at how bad the situation currently is. Predictions that globally we’re losing insect biodiversity at 9% per year should warn us. Milman argues that conservationists (rightly so, by the way) do all possible to stop tigers, rhino’s and orang-utans from going extinct, yet the downfall of small critters that are responsible for pollinating our fruits and vegetables is ignored, and their disappearance will have far more-reaching consequences for our own survival. In fact, our global food security will be directly at stake. It is therefore also our responsibility, as a malaria community, to safeguard this from happening in spite of our well-intended actions to save lives when using chemical pesticides.

Entire generations of malaria professionals have grown up not knowing better than that chemical pesticides are key in our (limited) arsenal of vector control tools. Many careers have been built around these tools and in some Universities entire research groups focus on the study of chemical pesticides. The Innovative Vector Control Consortium (or IVCC, based in Liverpool) has overwhelmingly focused on chemical insecticide discovery, valorisation, registration, and market entry, with both its former and current CEO having worked for decades for the chemical company Bayer. Chemical pesticides is their *raison d’être*, and making them change this course is hard to imagine. The same applies to chemical companies (Bayer, BASF, Sumitomo, Syngenta, etc.) that all have a (commercial, albeit limited in comparison to agricultural pesticides) stake in malaria vector control, but solely based on chemistry. Having them move away from chemistry is having them removed from their mission statements. And so they (likely) won’t - no matter how much they may appreciate (and even acknowledge) concerns over the negative impacts of their chemicals. Nevertheless, something needs to happen.

## Legislation, donors, and ethics

If the Global Fund would close the tap for funding of certain chemical pesticides or types of insecticide-treated nets, these would quickly disappear from being used by malaria-endemic countries. In turn, since the Global Fund will only allow products prequalified by WHO to have funds requested for by endemic countries, WHO has an equally important role to play. It can also restrict the use of certain chemicals and thus prevent their use in endemic settings. Next, donor funding can be highly influential: If the Gates Foundation (or UKAID, USAID, etc.) would support more environmentally-benign and sustainable tools for malaria vector control, IVCC would have to change its course. It’s the hand that feeds you that matters, so donors would equally benefit from some serious introspection regarding the (negative) consequences of the money they dish out. The US President’s Malaria Initiative (PMI) is already shying away from IRS in parts of Africa, having to adhere to US-EPA regulations. In essence a repetition of phasing out the use of DDT in Africa, following its ban in the USA in the early 1970s.

Underneath all of this lies a much more fundamental and ethical question: If certain chemicals are banned in the EU because of their known and acknowledged risks, why are we using these same chemicals in houses of African people? Clothianidin was banned for outdoor use in the EU as far back as 2018, based on a thorough review by EFSA [11], but WHO endorsed that same chemical in that same year for use in IRS, as well as another product based on this chemical in 2021 [12]. And as mentioned above, these chemicals will end up in the outdoor environment. Imidacloprid (another neonicotinoid) underwent the same fate in the EU (banned for outdoor use) but was endorsed by WHO for outdoor space spraying in 2019. Is the health of humans and the environment in low- and middle income countries less valuable than that of people living in the EU? Surely not, I hope to believe.

## Alternatives, they’re there but not exploited

We could simply stop using chemicals in bednets, albeit that this would not solve the waste problem. Although the benefits of treated over non-treated nets have been studied extensively and documented well, some argue that high-quality and strong untreated nets could be similar in offering good protection [13]. A net which doesn’t easily become torn and which is big enough to avoid direct contact with body parts offers adequate personal protection. Bite prevention rather than lethality of nets can deliver good public health value. In conjunction with the upcoming spatial repellents (I know, these are again more chemicals going into the environment), which are meant to stop mosquitoes from entering houses in the first place, untreated nets may find their niche. However, the chance that the oligarchy of large bednet manufacturers would even remotely consider this as an option is infinitely small. Regardless, considering the already wide-spread and intensifying resistance to pyrethroids, which will ultimately happen to all other chemicals used for bednet treatment, and very minimal chances of finding (safe) new actives to treat nets in future, may ultimately force us all to look for alternative ways to use nets.

The same applies to chemicals used for IRS - we will lose them all. If not sooner than later, simply based on the principles of evolution. Unless, maybe, when we start to use evolutionary principles ourselves against our mosquito enemy. The principles and advantages of late-in-life killing actives have been clearly outlined but have not been given serious attention. Give a female mosquito the chance to reproduce one or more batches of eggs before killing her after exposure to an active and the risk of resistance developing is significantly reduced (if not nil) [14].

Alternatively, we could use living organisms against our mosquito enemy, and co-evolve them to make sure that they keep the upper hand. Entomopathogenic fungi have been studied extensively against anophelines (in the lab, semi-field and open field) and clearly have big potential [15-19]. Seed batches of spores used for mass production can be selected on the basis of virulence, thereby preventing the rise of any form of resistance. Moreover, given that these fungi produce a suite of compounds upon entering the mosquito, the chances of mutants popping up that are resistant against all of these compounds is negligibly small. Critics argue that the residual lifespan of spores applied under tropical conditions is not long enough, but studies in Tanzania have shown that this is longer for fungi than for the WHO prequalified bendiocarb. Moreover, proper investment in strain selection (or even adaptation to survive under warmer and drier conditions) and formulation of spores can surely expand this period to reach desired levels (now considered to be six months for chemicals).

Genetic control of vectors have the huge advantage that they work without chemicals and are species-specific. Apart from classical SIT (Sterile Insect Technique [20]), however, genetic modification of mosquitoes for whatever trait, brings a suite of regulatory and ethical issues that need to be considered. The first proposals to release engineered mosquitoes to combat malaria date back thirty years [21]. Yet, in spite of significant advances in this field (including the more recent gene drive options [22]), such mosquitoes have yet to prove their merit in the fight against malaria. Oxitec’s releases of engineered mosquitoes to control malaria and the spread of *Anopheles stephensi* in Djibouti are so far a success in PR terms, but the true impact in epidemiological terms and elimination of this invasive vector in the Horn of Africa remains to be seen.

Odour-baited traps have been researched for decades, and although good attractant formulations for African anophelines have been developed [23], we currently lack cheap hardware (the trap itself) to put these to good use *en masse*. Yet, I’m convinced that when mass produced, the cost of such devices can fall below the price of two or three bednets and certainly be cost-competitive with IRS. Traps have been successfully used to control malaria in Kenya [24] and Ethiopia [25] and against dengue vectors in Asia [26,27], so there is no reason, *a priori*, to doubt their potential. The same argument can be made for eave tubes, which have shown epidemiological merit in an RCT conducted in Ivory Coast [28], for which now the first steps are made to replace the insecticide component with electrocuting grids, doing away with the need to use insecticides [29]. This latter development is a perfect example of how the transition from insecticidal to non-insecticidal approaches can and should materialise.

The list of potential alternatives is actually longer and includes exciting work on microsporidia [30]. Larval source management (LSM) with proven efficacy in numerous countries, is still not exploited in full, and will hopefully receive more attention in future [31]. Biological control agents for larvae (such as *Bti*) have been around for decades, and the more recent monomolecular films are showing great promise [32,33].

All of the above-mentioned alternatives (and this list is not even exhaustive) deserve more attention, and it is my hope that WHO, chemical companies and donors will admit that in order to avert Alonso’s crisis, we need to start doing things differently. None of these changes will happen overnight, but a clear plan is needed that will support malaria-endemic countries to move towards structural resilience, i.e., judiciously use chemical approaches but gradually phase these out in favour of environmentally-sound and sustainable approaches.

Recently, in another one of my posts on LinkedIn, in which I argued against aerial spraying (with chemicals) for malaria vector control in Africa, someone commented by calling me a tree hugger. It is my hope that this tree will flower next year, and that there will be enough insects left to pollinate it to ensure its survival. And produce fruit, and with that, our own survival.

## Conclusions

Around the world, insect biodiversity and abundance is decreasing at an alarming rate. Chemical pesticides play a significant role in this. Given that malaria vector control is largely based on the use of chemical pesticides, through bednets or indoor residual spraying, this decline in biodiversity is directly influenced by what those in malaria vector control use. It is likely, but remains unknown, that IRS and bednets (waste) have a direct impact on populations of non-target insects and this deserves to be studied properly. However, given that several of the chemicals in use in malaria vector control have been banned for use in the EU already signifies their danger when present in the environment for human, animal, or environmental health. Donors as well as international organisations like WHO and the Global Fund should play an active role in this. More importantly, there are biological alternatives, with proven efficacy, that could reach the market quickly if properly supported. In agriculture, this transition from chemical to biological control is much more advanced [34], and it is high time that the vector control field follows suit.
